# Supplementary material for: Multiplex translaminar imaging in the spinal cord of behaving mice
Source: Nat Commun. 2023 Mar 21;14:1427. doi: 10.1038/s41467-023-36959-2 (PMC10030868; doi:10.1038/s41467-023-36959-2)
Supplement: Supplementary file 1 — Supplementary Information [file 41467_2023_36959_MOESM1_ESM.pdf]

SUPPLEMENTARY INFORMATION

Multiplex translaminar imaging in the spinal cord of behaving mice

Pavel Shekhtmeyster[1,2,#], Erin M. Carey[1,#], Daniela Duarte[1], Alexander Ngo[1], Grace Gao[1], Nicholas A. Nelson[1,3], Charles L. Clark[1], and Axel Nimmerjahn[1,*]

[1]Waitt Advanced Biophotonics Center, The Salk Institute for Biological Studies, La Jolla, CA 92037, USA; [2]Electrical and Computer Engineering Graduate Program, University of California, San Diego, La Jolla, CA 92037, USA; [3]Biological Sciences Graduate Program, University of California, San Diego, La Jolla, CA 92037, USA

[#]These authors contributed equally to this work.

*Correspondence: animmerj@salk.edu (A.N.)

Supplementary Figures

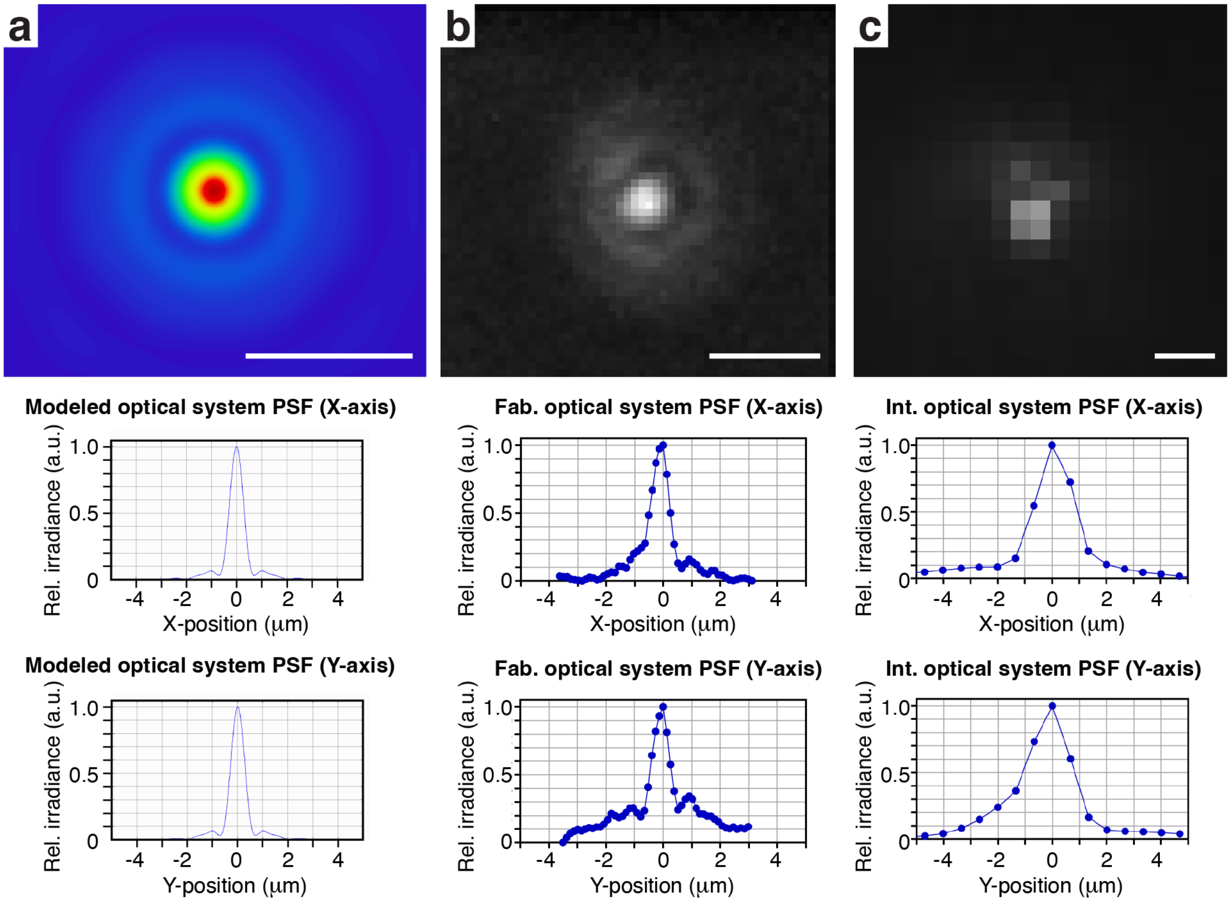

Supplementary Fig. 1 | Wearable microscopes with custom-compound microlenses provide <1.5 μm lateral resolution. a, *Top*, the image shows the optical system's lateral point spread function (PSF) as predicted by Zemax optical modeling. *Center*, x cross-section. *Bottom*, y cross-section. **b**, *Top*, experimentally measured lateral PSF of the fabricated optical system. *Center*, x cross-section. *Bottom*, y cross-section. **c**, *Top*, experimentally measured lateral PSF of the integrated microscope. *Center*, x cross-section. *Bottom*, y cross-section. Scale bars, 2 μm. All images are representatives from one sample. Images with similar properties were obtained across multiple independent samples.

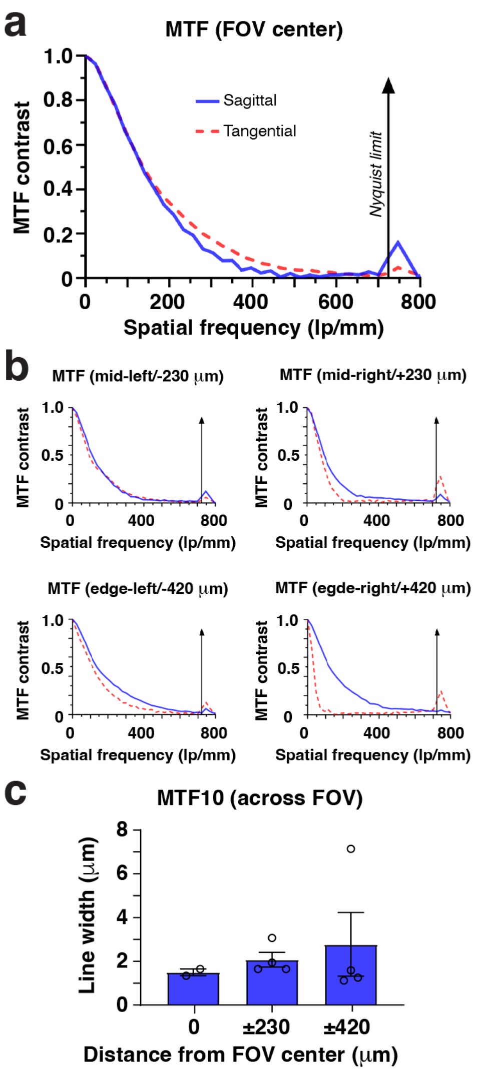

Supplementary Fig. 2 | Wearable microscopes with custom-compound microlenses provide high contrast across the field of view. a, Modulation transfer function (MTF) of the integrated microscope measured in the center of the field of view (FOV) using the Slanted Edge test. **b**, MTF at different indicated FOV positions relative to the center. **c**, MTF contrast at 10% (MTF10) across the FOV. Displayed values are averages across similar FOV locations and horizontal and vertical Slanted Edge targets. Spatial frequencies were converted to line widths. The data in c are from n=2 and n=4 measurements at 0 μm, and ±804 μm and ±1482 μm, respectively. The data are presented as mean ± s.e.m. The larger error bars toward the FOV edge likely indicate sample tilt. Source data are provided as a Source Data file.

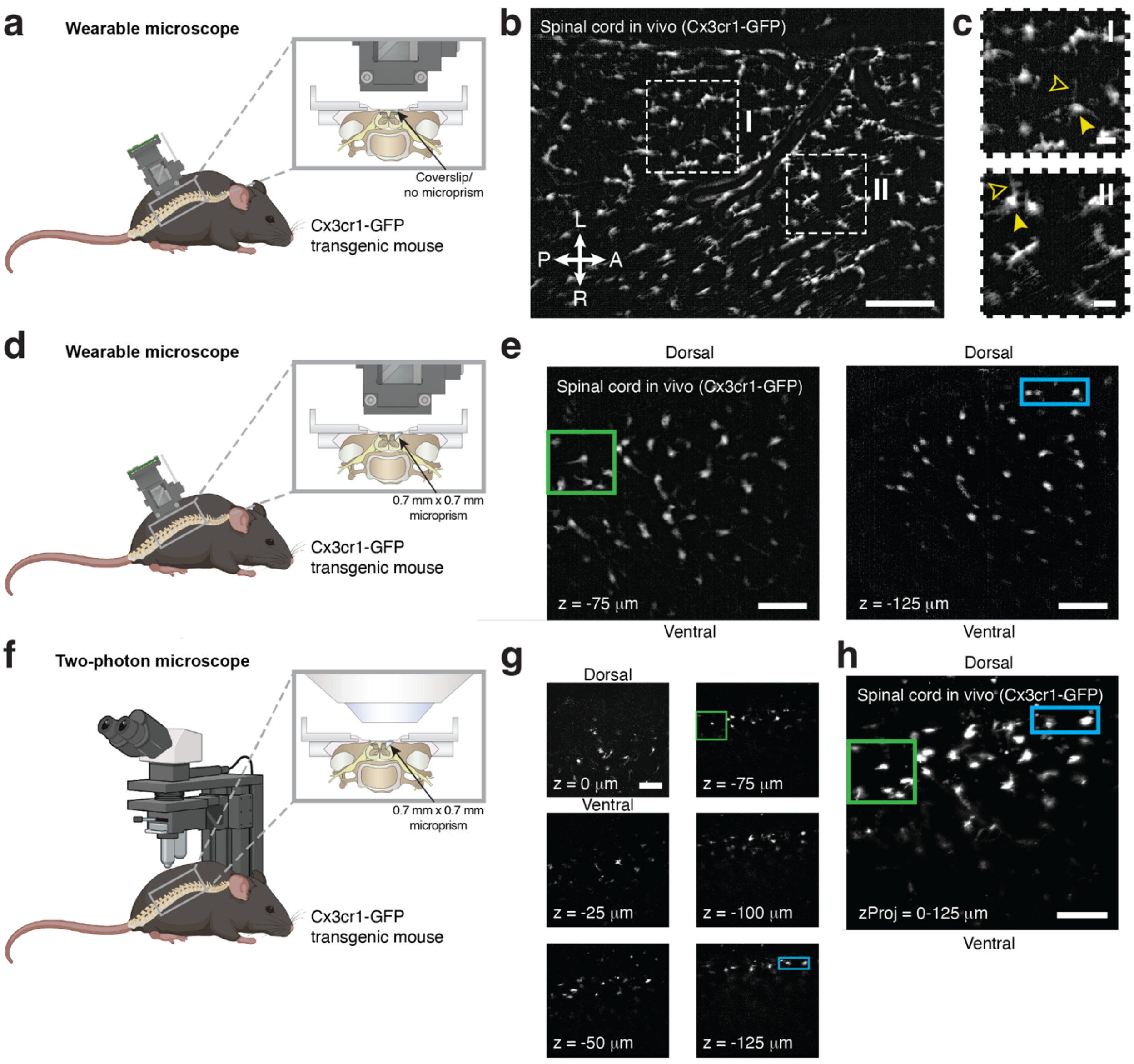

Supplementary Fig. 3 | See next page for caption.

Supplementary Fig. 3 | High-resolution intra- and translaminar imaging in Cx3cr1-GFP mice. **a**, Schematic of the intralaminar recording approach. A Cx3cr1-GFP mouse with GFP-labeled microglia was implanted with a lumbar dorsal glass window and then imaged under anesthesia with the wearable microscope. **b**, Fluorescence image showing microglial cells in the superficial spinal dorsal horn. Scale bar, 150 µm. **c**, Zoom-ins of the two subregions indicated in b. Individual microglial cell bodies and their fine processes (closed and open arrowheads, respectively) can be resolved. Scale bars, 25 µm. **d** and **f,** Schematics of the translaminar one- and two-photon recording approach. A Cx3cr1-GFP mouse with GFP-labeled microglia was implanted with a glass reflective microprism attached to a coverslip and then imaged with both the wearable (d-e) and a two-photon microscope (f-h) under anesthesia. **e,** Fluorescence images showing microglial cells across dorsal horn laminae. The images were acquired with the wearable microscope at 75 µm (left) and 125 µm focal depths (right). Scale bars, 100 µm. **g,** Example images from a two-photon z-stack of the same tissue region as in e acquired with 1 µm step size. z=0 µm denotes the vertical microprism-tissue interface. Scale bar, 100 µm. **h,** Maximum-intensity projection image through the 125 µm-thick z-stack. Scale bar, 100 µm. **e, g,** and **h,** The green and blue boxes indicate microglial cells visible in both the one- and two-photon data and located at 75 µm and 125 µm focal depths from the vertical microprism-tissue interface, respectively. All images are representatives from one sample. Images with similar properties were obtained across multiple independent samples.

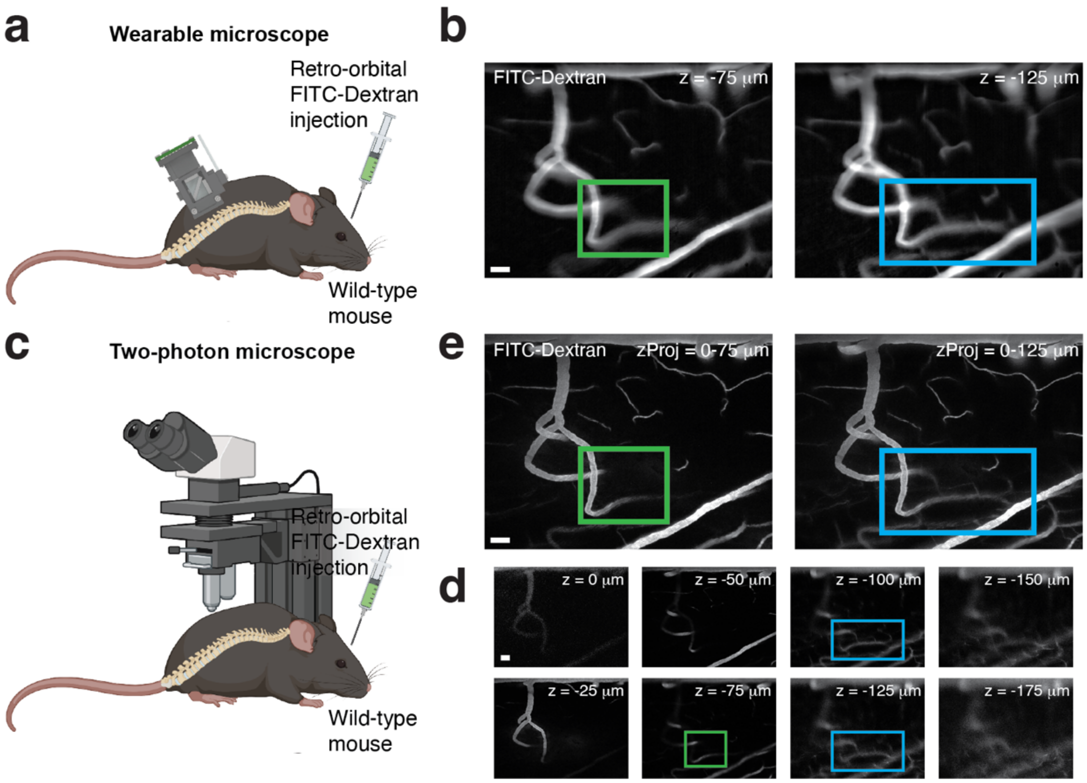

Supplementary Fig. 4 | Wearable microscopes with custom-compound microlenses allow spinal imaging up to ~125 µm depths with an extended depth-of-field. a and **c,** Schematics of the experimental approach. A wild-type mouse was prepared with a lumbar spinal window, injected retro-orbitally with FITC-Dextran (2% w/v), and imaged under anesthesia with both the wearable microscope (a-b) and a two-photon microscope (c-e). **b,** Example images from two time-lapse recordings (45 fps) acquired with the wearable microscope at 75 µm (left) and 125 µm focal depths (right) (**Supplementary Movie 1**). Each image is a 45-frame/1-s average. Scale bar, 50 µm. **d,** Example images from a two-photon z-stack acquired with 1 µm axial step size from the same spinal cord region as in b. z=0 µm denotes the tissue surface. Each image represents a 2-frame/1-s average. Scale bar, 50 µm. **e,** Maximum-intensity projection images over a 75 µm- (left) and 125 µm-thick tissue volume (right). Scale bar, 50 µm. **b**, **d**, and **e**, Comparison of the one- and two-photon images reveals that the wearable microscope permits imaging up to 125 µm depths with an extended depth-of-field, promoting stable recordings from dynamic or curved tissues, such as the spinal cord. The green and blue boxes indicate example microvessels seen in both the one- and two-photon data at 75 µm and 125 µm focal depths, respectively. All images are representatives from one sample. Images with similar properties were obtained across multiple independent samples.

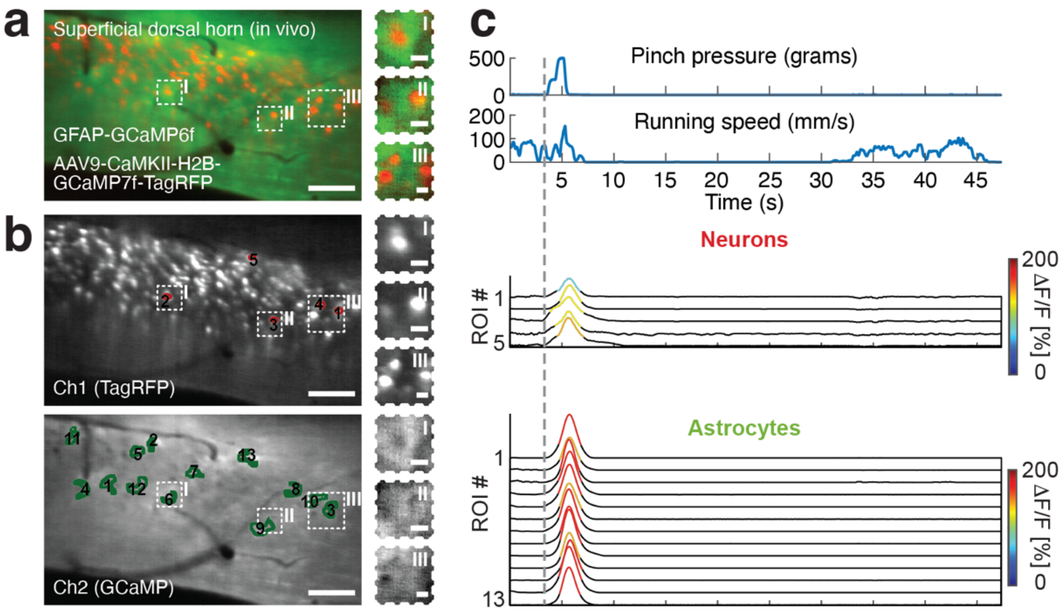

Supplementary Fig. 5 | Wearable microscopes with custom-compound microlenses permit multi-color imaging with a single image sensor. a, *Left*, example multi-color fluorescence image from the time-lapse recording of **Fig. 2e** showing neuronal nuclei (red) and surrounding astrocytes (green) in the spinal dorsal horn of a behaving mouse. *Right*, zoom-ins of the three subregions indicated on the left. Multiplex imaging was performed ~2.5 weeks after AAV9-CaMKII-H2B-GCaMP7f-TagRFP injection into the lumbar spinal cord of GFAP-GCaMP6f mice. Scale bars, 100 µm (left) and 15 µm (right). **b**, *Left*, color-separated images with neuronal (red) and astrocyte (green) regions of interest (ROIs) highlighted. *Right*, zoom-ins of the indicated subregions. Scale bars, 100 µm (left) and 15 µm (right). **c**, Noxious tail pinch-evoked neuronal nuclear and astrocyte calcium transients in the ROIs shown in b (**Supplementary Movie 2**). The corresponding pressure stimulus and locomotor activity are shown above the line traces. Running speed was recorded by placing the animal on a spherical treadmill. The vertical dashed line indicates pinch onset. All images are representatives from one sample. Images with similar properties were obtained across multiple independent samples.

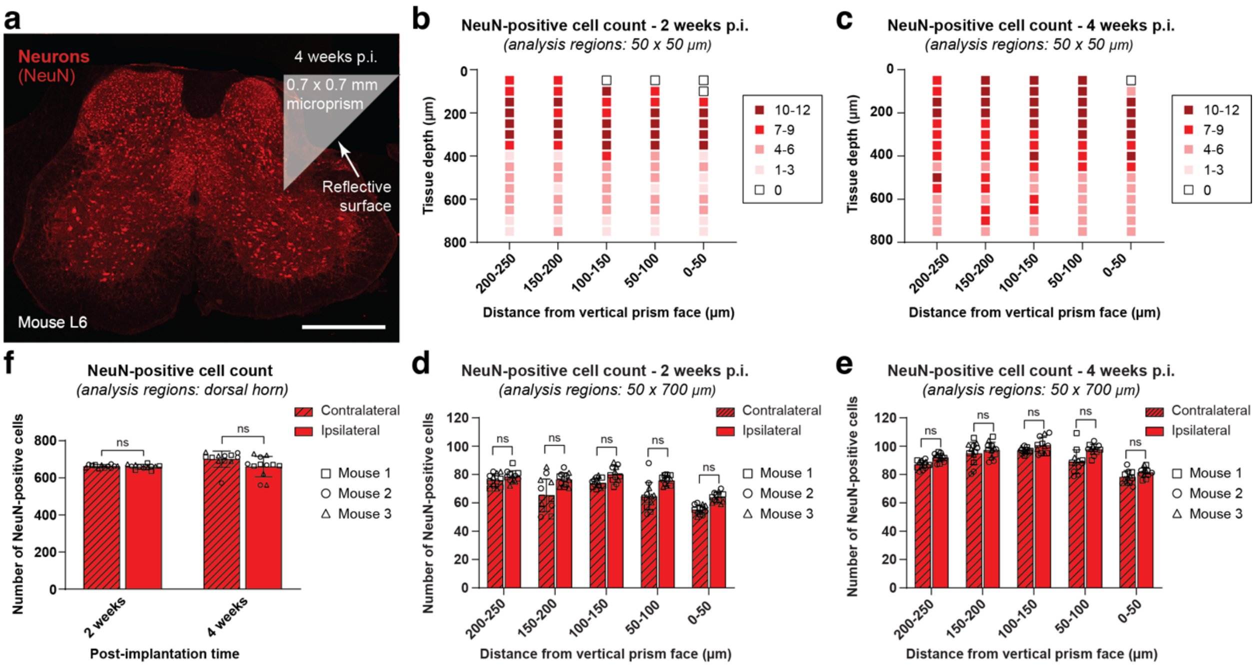

Supplementary Fig. 6 | Microprism implantation does not result in a significant loss of dorsal horn neurons. **a**, Fluorescence image showing NeuN-positive cells (neurons) in a 20-μm-thick mouse spinal cord section four weeks after microprism implantation. The approximate microprism location is indicated. Scale bar, 500 μm. **b-c**, Average NeuN-positive cell count in 50 μm x 50 μm analysis regions plotted as a function of tissue depth and distance from the vertical microprism face two (b) and four (c) weeks after microprism implantation. Cell density decreases with depth because of fewer cells with larger cell bodies. Depending on white matter thickness, cell density may also decrease toward the vertical microprism face. **d-e**, Average NeuN-positive cell count in 50 μm x 700 μm strips plotted as a function of distance from the vertical microprism face two (d) and four (e) weeks after microprism implantation. The data are compared to equivalent regions on the non-implanted (contralateral) hemisphere. **f**, Average NeuN-positive cell count across the entire dorsal horn two and four weeks after microprism implantation. The data are compared to equivalent regions on the non-implanted (contralateral) hemisphere. The data in b-f are from 12 slices and 3 mice for each time point. Two-sided paired t-tests determined *P* values, and all data are presented as mean ± s.e.m. The image in a is from one sample. Images with similar properties were obtained across multiple independent samples. Source data are provided as a Source Data file.

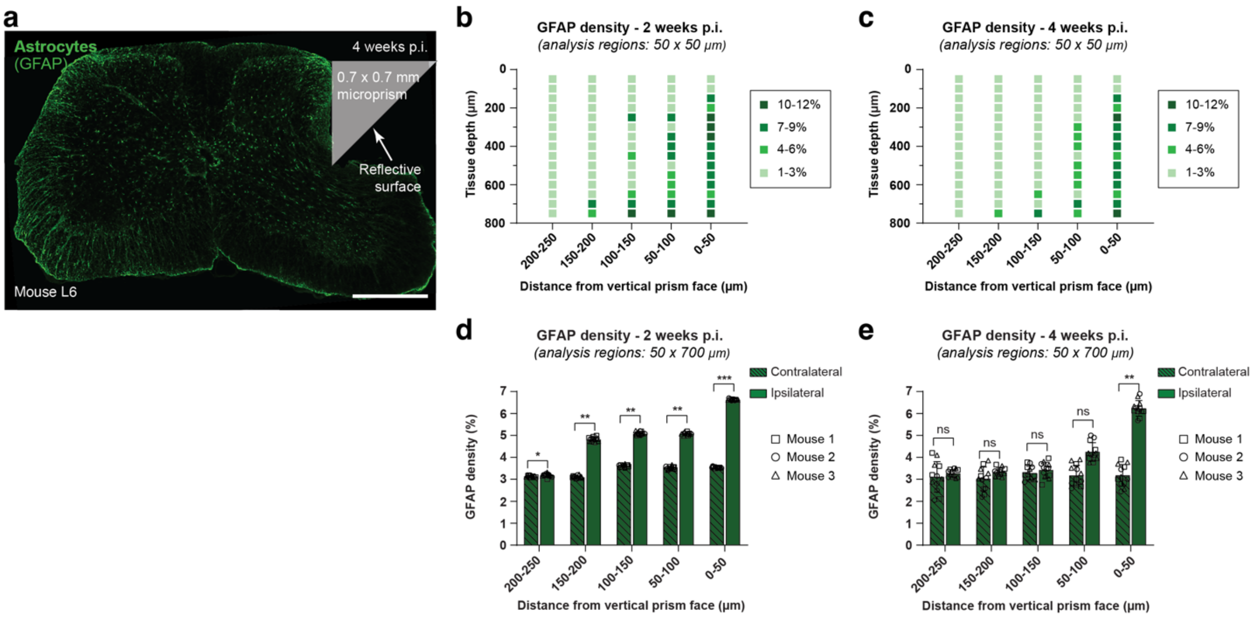

Supplementary Fig. 7 | Microprism implantation leads to a transient glial fibrillary acidic protein (GFAP) upregulation near the microprism-tissue interface. **a**, Fluorescence image showing GFAP staining (astrocytes) in a 20-μm-thick mouse spinal cord section four weeks after microprism implantation. The approximate microprism location is indicated. Scale bar, 500 μm. **b-c**, Average GFAP density in 50 μm x 50 μm analysis regions plotted as a function of tissue depth and distance from the vertical microprism face two (b) and four (c) weeks after microprism implantation. GFAP immunoreactivity decreases over time and is highly localized to regions immediately adjacent to the vertical microprism-tissue interface. **d-e**, Average GFAP density in 50 μm x 700 μm strips plotted as a function of distance from the vertical microprism face two (d) and four (e) weeks after microprism implantation. The data are compared to equivalent regions on the non-implanted (contralateral) hemisphere. The data in b-e are from 12 slices and 3 mice for each time point. Two-sided paired t-tests determined *P* values, and all data are presented as mean ± s.e.m. The image in a is from one sample. Images with similar properties were obtained across multiple independent samples. Source data are provided as a Source Data file.

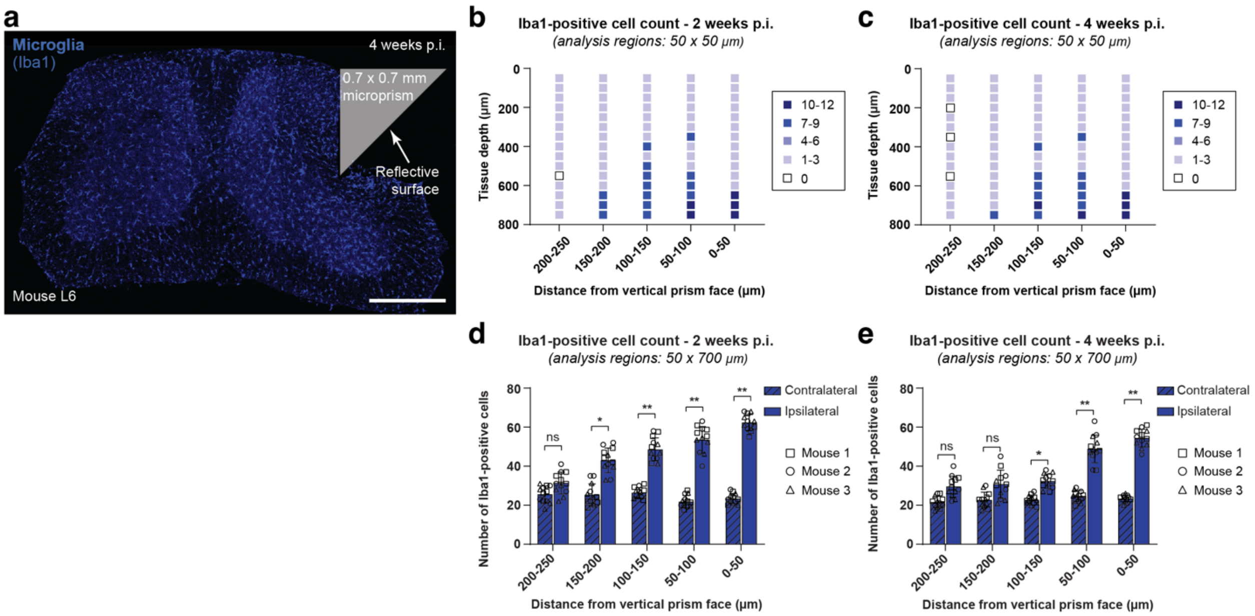

Supplementary Fig. 8 | Microprism implantation leads to a transient and highly localized Iba1 upregulation near the implanted microprism edge. **a**, Fluorescence image showing Iba1-positive cells (microglia/macrophages) in a 20-μm-thick mouse spinal cord section four weeks after microprism implantation. The approximate microprism location is indicated. Scale bar, 500 μm. **b-c**, Average Iba1-positive cell count in 50 μm x 50 μm analysis regions plotted as a function of tissue depth and distance from the vertical microprism face two (b) and four (c) weeks after microprism implantation. Iba1-positive cell count decreases over time and is highly localized to regions surrounding the implanted microprism edge. **d-e**, Average Iba1-positive cell count in 50 μm x 700 μm strips plotted as a function of distance from the vertical microprism face two (d) and four (e) weeks after microprism implantation. The data are compared to equivalent regions on the non-implanted (contralateral) hemisphere. The data in b-e are from 12 slices and 3 mice for each time point. Two-sided paired t-tests determined *P* values, and all data are presented as mean ± s.e.m. The image in a is from one sample. Images with similar properties were obtained across multiple independent samples. Source data are provided as a Source Data file.

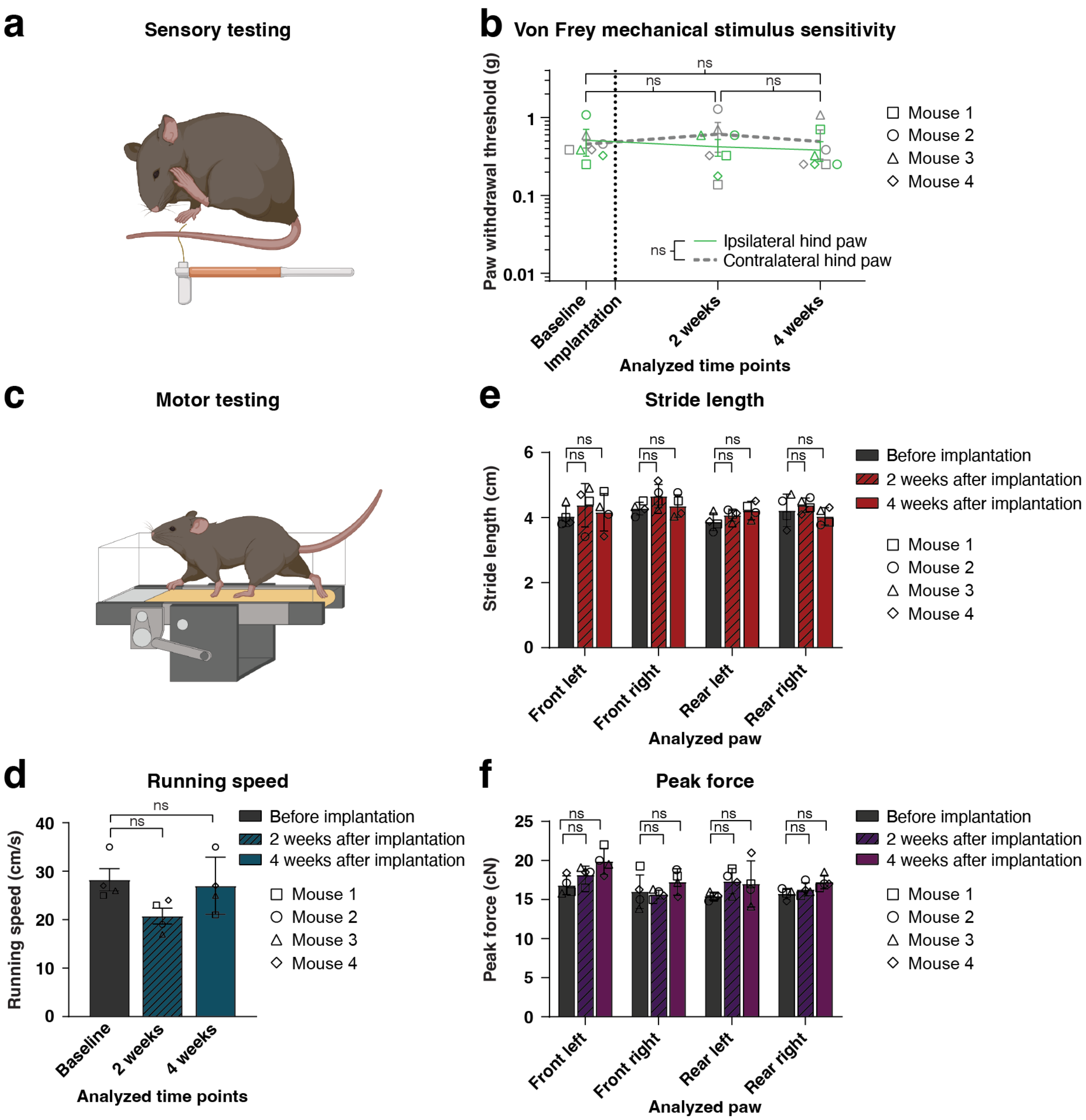

Supplementary Fig. 9 | See next page for caption.

Supplementary Fig. 9 | Microprism implantation does not result in overt sensory or motor deficits. **a**, Schematic showing the von Frey assay used for sensory testing. Hind paw withdrawal threshold was quantified for the implanted (ipsilateral) and non-implanted (contralateral) side before and at different time points after microprism implantation at the L4-L5 spinal level. **b**, Hind paw withdrawal threshold on the implanted (ipsilateral) and non-implanted (contralateral) side before, two, and four weeks after microprism implantation. Paw withdrawal threshold was not significantly different between time points and hemispheres (N=4 mice; ipsilateral baseline: 0.511 ± 0.193; two-week time point: 0.422 ± 0.103; four-week time point: 0.383 ± 0.108). *P* values were determined by Bonfierri-Dunn corrected two-sided paired t-tests between time points for the ipsilateral paw ($P > 0.05$) and two-way ANOVA across all time points between the contralateral and ipsilateral hind paws ($P = 0.62$). The average value of two test sessions is reported for each animal at each indicated time point (**Methods**). **c**, Schematic of the kinematic weight-bearing test used for motor testing. **d-f**, Population data showing running speed (d), front and hind paw stride length (e), and front and hind paw peak force (f) before, two, and four weeks after microprism implantation at the L4-L5 spinal level. Locomotor performance was not significantly different between time points (N=4 mice). All animals were tested at each indicated time point (**Methods**). Two-sided paired t-tests determined *P* values, and all data are presented as mean ± s.e.m. Source data are provided as a Source Data file.

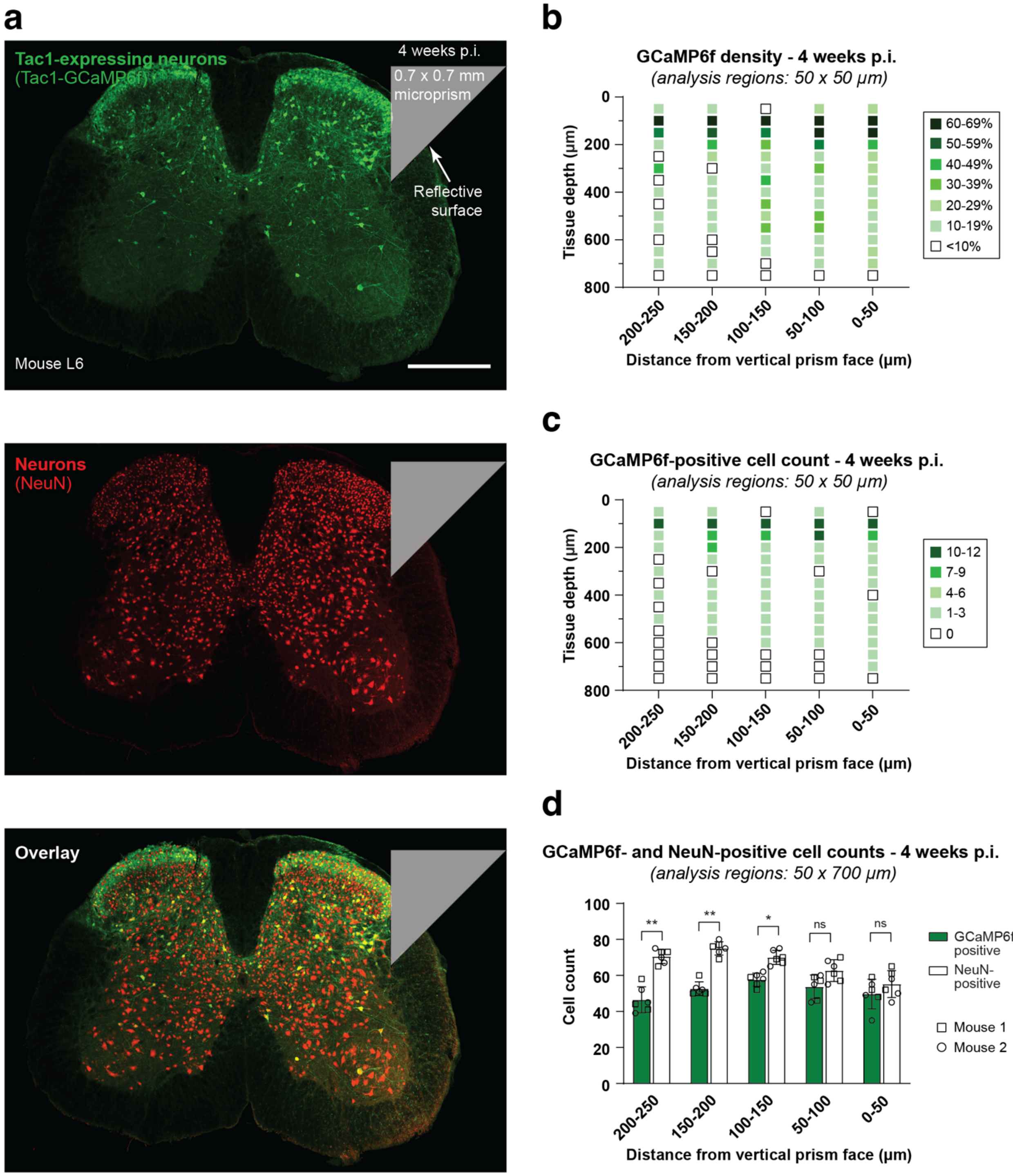

Supplementary Fig. 10 | See next page for caption.

Supplementary Fig. 10 | Distribution of calcium indicator-expressing neurons in the lumbar dorsal horn of Tac1-GCaMP6f mice. a, Fluorescence images showing GCaMP6f- (top) and NeuN-positive cells (center) in a 20-µm-thick spinal cord section from a Tac1-GCaMP6f mouse four weeks after microprism implantation. *Bottom*, overlay of the top and center images. The approximate microprism location is indicated. Scale bar, 250 µm. **b-c**, Average GCaMP6f density (b) and GCaMP6f-positive cell count (c) in 50 µm x 50 µm analysis regions plotted as a function of tissue depth and distance from the vertical microprism face four weeks after microprism implantation (**Methods**). While calcium indicator-expressing neurons in Tac1-GCaMP6f mice are concentrated in superficial laminae, cell bodies and processes are found throughout the optically accessible dorsoventral (up to 700 µm depth) and mediolateral tissue regions (up to ~125 µm distance from the vertical tissue-microprism interface) (**Fig. 4c**; **Supplementary Fig. 3e**). **d**, Average GCaMP6f- and NeuN-positive cell counts in 50 µm x 700 µm strips plotted as a function of distance from the vertical microprism face four weeks after microprism implantation. The data in b-d are from 6 slices and N=2 mice. Two-sided paired t-tests determined *P* values, and all data are presented as mean ± s.e.m. All images are representatives from one sample. Images with similar properties were obtained across multiple independent samples. Source data are provided as a Source Data file.

Supplementary Tables

Surface	Type	Radius	Thickness	Glass	Diameter
OBJ	STANDARD	Infinity	0.0000000		6.0000000
1	STANDARD	Infinity	4.5000000		4.8000000
2	STANDARD	-13.65635	1.0000000	N-SF57	2.6790000
3	STANDARD	1.758689	0.9479822		2.3490000
4	STANDARD	-10.38426	1.0000000	N-BALF5	2.6570000
5	STANDARD	9.049044	1.1821360	N-LASF40	3.9880000
6	STANDARD	-3.253426	0.099919		3.7850000
7	STANDARD	Infinity	6.5140000	FSILICA	4.2000000
8	STANDARD	Infinity	0.0000000		4.2000000
9	STANDARD	Infinity	0.408000		2.5160000
STO	STANDARD	5.543071	1.0000000	N-SF66	2.5160000
11	STANDARD	1.973004	1.1777090	N-PK51	2.5160000
12	STANDARD	-5.628253	0.1000000		2.9940000
13	STANDARD	2.635428	1.1278330	N-LAK34	3.2970000
14	STANDARD	-13.04288	2.0000000		3.3600000
15	STANDARD	Infinity	0.1000000	D263M	1.0320000
IMA	STANDARD	Infinity			0.9784000

Supplementary Table 1 | Wearable microscope optical design. The specified values reflect the optical model parameters.
