# Supplementary material for: Multiplex translaminar imaging in the spinal cord of behaving mice
Source: Nat Commun. 2023 Mar 21;14:1427. doi: 10.1038/s41467-023-36959-2 (PMC10030868; doi:10.1038/s41467-023-36959-2)
Supplement: Supplementary file 2 — Description of Additional Supplementary Files [file 41467_2023_36959_MOESM2_ESM.pdf]

Description of Additional Supplementary Files

Supplementary Movie 1: **High-speed blood flow imaging in the spinal cord of an anesthetized FITC-Dextran-injected wild-type mouse**. Example time-lapse recordings (45 fps) acquired with the wearable microscope at 75 μm (left) and 125 μm focal depths (right) in an anesthetized wild-type mouse, injected retro-orbitally with FITC-Dextran (2% w/v). Imaging was performed through a dorsal optical window over the lumbar spinal cord (**Supplementary Fig. 4a-b**). Scale bar, 50 μm. While contrast and resolution are expected to degrade with imaging depth (**Fig. 3**), blood flow remains traceable in small capillaries in vivo.

Supplementary Movie 2: Multiplex intralaminar imaging of tail pinch-evoked calcium activity in spinal astrocytes and neurons of a behaving mouse. *Top*, example multi-color fluorescence time-lapse recording of **Fig. 2e-g** showing neuronal nuclei (red) and surrounding astrocytes (green) in the spinal dorsal horn of a behaving mouse. Multiplex imaging through a dorsal optical window was performed ~2.5 weeks after AAV9-CaMKII-H2B-GCaMP7f-TagRFP injection into the lumbar spinal cord of a GFAP-GCaMP6f mouse. Scale bar, 100 μm. *Center* and *bottom*, color-separated images. Sparse nuclear calcium transients in local excitatory neurons precede widespread astrocyte excitation (**Supplementary Fig. 5c; Discussion**).

Supplementary Movie 3: High-speed translaminar imaging of tail pinch-evoked calcium activity in the spinal cord of a behaving GFAP-GCaMP6f mouse. *Top left*, example behavior video showing a GFAP-GCaMP6f mouse with the wearable microscope on a spherical treadmill. This experimental approach facilitates the precise placement of sensory stimuli (e.g., tail pinch) and provides a quantitative readout of locomotor activity. *Bottom left*, simultaneously acquired time-lapse recording (~42.3 fps) showing noxious tail pinch-evoked calcium excitation in spinal astrocytes (**Fig. 4d-e**). The data were obtained at ~75 μm focal depth from the vertical tissue-microprism interface four weeks after microprism implantation. Elapsed time is indicated in the upper right corner. Note that despite the dense tissue labeling, cellular-size large-amplitude transients can be seen throughout the field of view. *Right*, zoom-ins of the three indicated subregions on the bottom left. Noxious pinch triggered coordinated calcium excitation in the central field of view regions corresponding to upper dorsal horn laminae (**Fig. 4a-b**). Some activation in lower parts of the FOV is also seen, likely due to the accompanying brief motor response. Scale bars, 100 μm (bottom left) and 50 μm (right).

Supplementary Movie 4: High-speed translaminar imaging of motor-evoked calcium activity in the spinal cord of a behaving GFAP-GCaMP6f mouse. *Top left*, example behavior video showing a GFAP-GCaMP6f mouse with the wearable microscope on a spherical treadmill. This experimental approach provides a quantitative readout of locomotor activity. *Bottom left*, simultaneously acquired time-lapse recording (~40.3 fps) showing running-evoked calcium excitation in spinal astrocytes (**Fig. 4i-j**). The data were obtained at ~75 μm focal depth from the vertical tissue-microprism interface four weeks after microprism implantation. Elapsed time is indicated in the upper right corner. Despite the dense tissue labeling, cellular-size large-amplitude transients can be seen throughout the field of view. *Right*, zoom-ins of the three indicated subregions on the bottom left. Running triggered coordinated calcium excitation in the lower field of view regions likely corresponding to spinal premotor areas (**Fig. 4a-b**). Scale bars, 100 μm (bottom left) and 50 μm (right).

Supplementary Movie 5: High-speed translaminar imaging of tail pinch-evoked calcium activity in the spinal cord of a behaving Tac1-GCaMP6f mouse. *Top left*, example behavior video showing a Tac1-GCaMP6f mouse with the wearable microscope on a spherical treadmill. This experimental approach facilitates the precise placement of sensory stimuli (e.g., tail pinch) and provides a quantitative readout of locomotor activity. *Bottom left*, simultaneously acquired time-lapse recording (~43.2 fps) showing noxious tail pinch-evoked calcium excitation in spinal Tac1-expressing neurons. The data were obtained at ~75 μm focal depth from the vertical tissue-microprism interface four weeks after microprism implantation. Elapsed time is indicated in the upper right corner. Despite the dense tissue labeling, cellular-size large-amplitude transients can be seen throughout the field of view. *Right*, zoom-ins of the three indicated subregions on the bottom left. Noxious pinch triggered coordinated calcium excitation in the central field of view regions corresponding to upper dorsal horn laminae (**Fig. 4a-b**). Scale bars, 100 μm (bottom left) and 50 μm (right).
