# Supplementary material for: Multiplex translaminar imaging in the spinal cord of behaving mice
Source: Nat Commun. 2023 Mar 21;14:1427. doi: 10.1038/s41467-023-36959-2 (PMC10030868; doi:10.1038/s41467-023-36959-2)
Supplement: Supplementary file 8 — Reporting Summary [file 41467_2023_36959_MOESM8_ESM.pdf]

nature portfolio

Corresponding author(s): Axel Nimmerjahn

Last updated by author(s): February 5, 2023

Reporting Summary

Nature Portfolio wishes to improve the reproducibility of the work that we publish. This form provides structure for consistency and transparency in reporting. For further information on Nature Portfolio policies, see our Editorial Policies and the Editorial Policy Checklist.

Statistics

For all statistical analyses, confirm that the following items are present in the figure legend, table legend, main text, or Methods section.

n/a	Confirmed	
☐	☒	The exact sample size (n) for each experimental group/condition, given as a discrete number and unit of measurement
☐	☒	A statement on whether measurements were taken from distinct samples or whether the same sample was measured repeatedly
☐	☒	The statistical test(s) used AND whether they are one- or two-sided<br>*Only common tests should be described solely by name; describe more complex techniques in the Methods section.*
☐	☒	A description of all covariates tested
☐	☒	A description of any assumptions or corrections, such as tests of normality and adjustment for multiple comparisons
☐	☒	A full description of the statistical parameters including central tendency (e.g. means) or other basic estimates (e.g. regression coefficient) AND variation (e.g. standard deviation) or associated estimates of uncertainty (e.g. confidence intervals)
☐	☒	For null hypothesis testing, the test statistic (e.g. F, t, r) with confidence intervals, effect sizes, degrees of freedom and P value noted<br>*Give P values as exact values whenever suitable.*
☒	☐	For Bayesian analysis, information on the choice of priors and Markov chain Monte Carlo settings
☒	☐	For hierarchical and complex designs, identification of the appropriate level for tests and full reporting of outcomes
☒	☐	Estimates of effect sizes (e.g. Cohen's d, Pearson's r), indicating how they were calculated

Our web collection on statistics for biologists contains articles on many of the points above.

Software and code

Policy information about availability of computer code

Data collection	Wearable microscope images were acquired using DevWare (Aptina; v4.5.18).<br>Analog data were acquired using DAQExpress 2.0 (National Instruments).<br>Video data of mice were acquired using a Stingray F-033 camera (Allied Vision Technologies) and AVT SmartView software (v1.11).<br>Two-photon image data were collected using MPScope software (Kleinfeld lab, UCSD; v1.1).<br>Confocal image data were acquired using ZEN Black (Zeiss; v2011).<br>Kinematic weight-bearing data were acquired using BIO-KWB (Bioseb; v1.2.5).
Data analysis	Optical modeling was performed in Zemax (Zemax LLC; v12 R2 2012).<br>Optomechanical parts were designed in CAD software (Autodesk Inventor Professional; v2018).<br>Tile-based image analysis was performed using custom Fiji/ImageJ scripts (SciJava; v2.0.0c).<br>In vivo imaging and analog data were analyzed using custom MATLAB code (MathWorks, Inc.; R2012b and R2019b).<br>Two-photon images were processed and analyzed using ImageJ (1.53f51).<br>Confocal data was analyzed using Imaris software (Oxford Instruments; v 9.2).<br>Statistical analysis was performed in Excel (Microsoft; v2013) and Prism (GraphPad; v8.4).<br>Figures and Movies were produced in Illustrator and Premier, respectively (Adobe; v2022).<br>The custom software/code generated during the current study is available from the corresponding author on request.

For manuscripts utilizing custom algorithms or software that are central to the research but not yet described in published literature, software must be made available to editors and reviewers. We strongly encourage code deposition in a community repository (e.g. GitHub). See the Nature Portfolio guidelines for submitting code & software for further information.

Data

Policy information about availability of data

All manuscripts must include a data availability statement. This statement should provide the following information, where applicable:

- Accession codes, unique identifiers, or web links for publicly available datasets
- A description of any restrictions on data availability
- For clinical datasets or third party data, please ensure that the statement adheres to our policy

Source Data files for data presented in graphs within the Figures and Supplementary Figures are provided with this paper. Additional (e.g., raw image) data that support the findings of this study are available from the corresponding author. Because of their size, they can only be shared on request.

Field-specific reporting

Please select the one below that is the best fit for your research. If you are not sure, read the appropriate sections before making your selection.

☒ Life sciences ☐ Behavioural & social sciences ☐ Ecological, evolutionary & environmental sciences

For a reference copy of the document with all sections, see nature.com/documents/nr-reporting-summary-flat.pdf

Life sciences study design

All studies must disclose on these points even when the disclosure is negative.

Sample size

Sample sizes were not pre-determined and are consistent with the sample sizes typically used for optical instrument characterization and in vivo biological measurements, including transgenic animal validation, behavioral assessment, and live animal optical recordings (Scott, B. B. et al. Imaging Cortical Dynamics in GCaMP Transgenic Rats with a Head-Mounted Widefield Macroscope; Rynes, M. L. et al. Miniaturized head-mounted microscope for whole-cortex mesoscale imaging in freely behaving mice; and Sekiguchi, K. J. et al. Imaging large-scale cellular activity in spinal cord of freely behaving mice). Sex as a biological variable was not considered in the research design and analyses, as the study's primary goal was to demonstrate the imaging approach's technical capabilities.
For experiments in which the technical capabilities of the macroscope were characterized, a sample size of only 1 was needed as no biological variables were being studied (Figs. 1 - 3; Supplementary Figs. 1-5).
For experiments in which animal behavior was measured, a sample size of 4 animals was used (Supplementary Fig. 9).
For experiments where immunostaining was quantified, a sample size of 2-3 animals was used (Supplementary Figs. 6-8 and 10).
For experiments measuring the calcium activity in either neurons or astrocytes of behaving mice, a sample size of 3 - 5 animals was used (Figs. 4-5).

Data exclusions

Multi-color intralaminar imaging (Fig. 2; Supplementary Fig. 5): Mice with weak viral vector expression or suboptimal surgical preparation were excluded from data analysis. Data for which image motion artifact correction failed were also excluded from the analysis. We excluded trials computationally that did not meet pre-established criteria (e.g., tail pinch stimuli <500 g or shorter and longer than 1-2 s).
Translaminar imaging through implanted microprisms (Figs. 4, 5): Animals that showed vascular growth near the coverslip or microprism interface, as characterized by static blood vessels in the field of view, were excluded from data analysis. One in five mice fell into this category. If image motion artifact correction failed, the data were excluded from the analysis. One in four animals fell into this category. We also excluded trials computationally that did not meet pre-established criteria (e.g., tail pinch stimuli <500 g or shorter and longer than 1-2 s, and runs shorter and longer than 2-4 s or <25 s rest period before the run).
Histology (Supplementary Figs. 6-8, 10): Slices were excluded from analysis if the staining was uneven/irregular.
Sensory testing (Supplementary Fig. 9): Typically, mice with a baseline sensitivity greater than two standard deviations from the mean during baseline testing are excluded. However, no mice were excluded from our von Frey experiments in the study at hand.
Motor testing (Supplementary Fig. 9): Trials in which the animal did not continuously run from left to right in the setup were excluded.

Replication	All experiments characterizing the microscope's technical capabilities were performed independently. For all experiments involving live animals, the results were replicated successfully within and/or across animals. All replication attempts with a successful surgical preparation were successful. For immunohistochemistry experiments, 2-3 biological replicates were used, and 3-4 slices per animal were quantified.<br>For experiments demonstrating the imaging approach's technical capabilities, images in the figures are representatives from one sample. Images with similar properties were obtained across multiple independent samples (Figs. 1-3 and Supplementary Figs. 1-4).<br>In vivo multi-color imaging (Fig. 2, Supplementary Fig. 5, and Supplementary Movie 2) was performed/replicated using 3 AAV9-CaMKII-H2B-GCaMP7f-TagRFP vector-injected GFAP-GCaMP6f mice. The shown data is representative of these 3 mice.<br>Translaminar imaging through implanted microprisms (Figs. 4-5 and Supplementary Movies 3-5): The population data are from 5 GFAP- and 3 Tac1-GCaMP6f mice. Multiple trials were recorded and averaged per animal (see Figure legends). The findings were reproducible across animals.<br>Blood vessel/flow imaging (Supplementary Fig. 4 and Supplementary Movie 1): The data is from 1 mouse but representative of 2 technical replicates, which were both successful.<br>Histology: The population data shown in Supplementary Figs. 6-8 are averages across three animals and four slices per animal at each time point (two and four weeks after microprism implantation). Supplementary Fig. 10 shows averages across 2 animals and three slices per animal.<br>Sensory testing (Supplementary Fig. 9): The population data are from four animals. Each time point represents the average of two sessions per animal.<br>Motor testing (Supplementary Fig. 9): The population data is an average across four animals and five runs per animal at each time point (before, two, and four weeks after microprism implantation).
Randomization	There were no treatment groups in this study. Thus, randomization was not relevant and was not used for most experiments.<br>Multi-color and translaminar imaging (Figs. 2, 4, 5): The order in which mechanical stimuli of different intensity (range: 50-1,100 g) was delivered to the animal's proximal tail region was randomized.<br>Sensory testing (Supplementary Fig. 9): The animals were placed into the testing chambers and recorded in random order (i.e., removed from their home cage before the researcher could visibly identify the ear tag number). This randomization removes potential biases from different test chambers in the behavior setup.<br>Motor testing (Supplementary Fig. 9): The animals were placed into the testing chamber before the researcher read the ear tag number. Once sufficient runs were collected, the animal was returned to its home cage, and the ear tag number was recorded.
Blinding	Blinding was not necessary for in vivo experiments because no treatment groups existed. Investigators could not be blinded during data collection because they had to monitor the animal's well-being and behavior during the experiment and apply a comparable set of stimuli to the animal. Analyses were carried out independently from the data collection for all experiments and automatically using custom analysis software/codes.<br>Multi-color and translaminar imaging (Figs. 2, 4, 5): The investigators were not blinded during data collection/analysis. However, all analysis steps were automated, relying on the objective judgment of the analysis software.<br>Sensory testing (Supplementary Fig. 9): One experimenter performed the surgical microprism implantation. A different experimenter performed the behavioral test. This experimenter was blinded during behavior testing to the spinal cord hemisphere in which the microprism was implanted.<br>Motor testing (Supplementary Fig. 9): The investigator was not blinded to the outcome assessment because they needed to record the animal identifier. However, all of the analysis steps were automated.

Reporting for specific materials, systems and methods

We require information from authors about some types of materials, experimental systems and methods used in many studies. Here, indicate whether each material, system or method listed is relevant to your study. If you are not sure if a list item applies to your research, read the appropriate section before selecting a response.

Materials & experimental systems

n/a	Involved in the study	
☐	☒	Antibodies
☐	☒	Eukaryotic cell lines
☒	☐	Palaeontology and archaeology
☐	☒	Animals and other organisms
☒	☐	Human research participants
☒	☐	Clinical data
☒	☐	Dual use research of concern

Methods

n/a	Involved in the study	
☒	☐	ChIP-seq
☒	☐	Flow cytometry
☒	☐	MRI-based neuroimaging

Antibodies

Antibodies used	The following primary antibodies were used:<br>Mouse monoclonal anti-NeuN/RBFOX3 clone 1B7 (Novus Biologicals Inc.; cat. no. NBP1-92693; RRID: AB_11036146)<br>Rat monoclonal anti-GFAP clone 2.2B10 (Thermo Fisher Scientific; cat. no. 13-0300; RRID: AB_2532994)<br>Rabbit polyclonal anti-Iba1 (FUJIFILM Wako Shibayagi; cat. no. 019-19741; RRID: AB_839504)<br>Chicken polyclonal anti-GFP (Thermo Fisher Scientific; cat. no. A10262; RRID: AB_2534023)<br><br>The following secondary antibodies were used:<br>Goat anti-mouse IgG(H+L), Alexa Fluor 633 (Thermo Fisher Scientific; cat. no. A-21052; RRID: AB_2535719)

	Goat anti-rat IgG(H+L), Alexa Fluor 488 (Thermo Fisher Scientific; cat. no. A-11006; RRID: AB_2534074)<br>Goat anti-chicken IgG(H+L), Alexa Fluor 488 (Thermo Fisher Scientific; cat. no. A-11039; RRID: AB_2534096)<br>Goat anti-rabbit IgG(H+L), Alexa Fluor 405 (Thermo Fisher Scientific; cat. no. A-31556; RRID: AB_221605)
Validation	Validation according to the manufacturer's website for:<br><br>a) anti-NeuN (Novus Biologicals Inc.; cat. no. NBP1-92693): "We have publications tested in 3 confirmed species: Human, Mouse, Rat. We have publications tested in 6 applications: Flow, ICC/IF, IF, IHC, IHC-Fr, IHC-P."<br><br>b) anti-GFAP (Thermo Fisher Scientific; cat. no. 13-0300): The manufacturer's website states that this antibody has been validated and referenced in IHC applications for 115 publications.<br><br>c) anti-Iba1 (FUJIFILM Wako Shibayagi; cat. no. 019-19741): "Iba1 (Ionized calcium-binding adapter molecule1) is an approximately 17 kDa calcium-binding protein. It is used as a microglial marker because it is expressed specifically in microglia in the central nervous system1). It is expressed in both resting and activated microglia, but is reportedly expressed more highly in activated microglia2). It is also expressed in macrophages in peripheral tissues and is known as AIF-1 (Allograft inflammatory factor-1). Iba1 binds to F-actin in cells to form actin bundles. The formation of actin bundles is thought to be required for the membrane ruffling observed during cell migration and phagocytosis3). FUJIFILM Wako's "Anti Iba1, Rabbit" (Product Number 019-19741), which allows even microglia processes to be stained by immunohistochemical staining, is used by researchers all over the world as a microglia marker antibody standard."<br><br>d) anti-GFP (Thermo Fisher Scientific; cat. no. A10262): The manufacturer's website states that this antibody has been validated and referenced in IHC applications for 192 publications. It also says, "This Antibody was verified by Relative expression to ensure that the antibody binds to the antigen stated."<br><br>e) Alexa Fluor 633 (Thermo Fisher Scientific; cat. no. A-21052): IHC, ICC, and flow cytometry on the manufacturer's website and IHC in this manuscript.<br><br>f) Alexa Fluor 488 (Thermo Fisher Scientific; cat. no. A-11006): ICC and flow cytometry on the manufacturer's website and IHC in this manuscript.<br><br>g) Alexa Fluor 488 (Thermo Fisher Scientific; cat. no. A-11039): IHC, ICC, and flow cytometry on the manufacturer's website and IHC in this manuscript.<br><br>h) Alexa Fluor 405 (Thermo Fisher Scientific; cat. no. A-31556): IHC, ICC, and flow cytometry on the manufacturer's website and IHC in this manuscript.

Eukaryotic cell lines

Policy information about cell lines

Cell line source(s)	293AAV Cell Line (Cell Biolabs, Inc.; cat. no. AAV-100)
Authentication	The 293AAV Cell Line is a permanent line established from primary embryonic human kidney transformed with human adenovirus type 5 DNA. The genes encoded by the E1 region of adenovirus (E1a and E1b) are expressed in these cells and participate in the transactivation of viral promoters, allowing these cells to produce high protein levels. 293AAV is derived from the parental 293 cell line through cloning and multiple rounds of testing. 293AAV is specifically selected for a high level of AAV production in a helper-free system. Statement of quality control: "This cryovial contains at least 1.0 × 10^6 293AAV cells as determined by morphology, trypan-blue dye exclusion, and viable cell count."
Mycoplasma contamination	From the datasheet: "The 293AAV cells are tested free of microbial contamination."
Commonly misidentified lines<br>(See ICLAC register)	No commonly misidentified cell lines were used in this study.

Animals and other organisms

Policy information about studies involving animals; ARRIVE guidelines recommended for reporting animal research

Laboratory animals	Mouse strains used in this study included GFAP-Cre (RRID: IMSR_JAX:012886), Tac1-Cre (RRID: IMSR_JAX:021877), Ai95(RCL-GCaMP6f)-D (RRID: IMSR_JAX:024105), heterozygous Cx3cr1-GFP (RRID: IMSR_JAX:005582), and wild-type mice. All experiments involved heterozygous male and female mice, 6-11 weeks old. All mice were on a C57BL/6J background. Sex as a biological variable was not considered in the research design and analyses, as the study's primary goal was to demonstrate the imaging approach's technical capabilities.<br><br>21 GFAP-GCaMP6f mice were used:<br>3 females, 8-11 weeks old, were used for multi-color in vivo imaging (Fig. 2, Supplementary Fig. 5, Supplementary Movie 2).<br>5 females, 11 weeks old, were used for in vivo imaging through spinal cord-implanted microprisms (Fig. 4 and Supplementary Movies 3 and 4).<br>6 females, 9 or 11 weeks old, were used for histology (3 each for the two- and four-week time points) (Supplementary Figs. 6-8).<br>3 females, 7 weeks old, were used for von Frey behavior testing (Supplementary Fig. 9).<br>4 females, 7 weeks old, were used for kinematic weight-bearing testing (Supplementary Fig. 9).

6 Tac1-GCaMP6f mice were used:
3 females, 11 weeks old, were used for in vivo imaging through spinal cord-implanted microprisms (Fig. 5 and Supplementary Movie 5).
1 female, 7 weeks old, was used for von Frey behavior testing (Supplementary Fig. 9).
1 male and 1 female, each 11 weeks old, were used for histology (Supplementary Fig. 10).

2 Cx3cr1-GFP mice were used:
2 females, 8 weeks old, were used for in vivo intra- and translaminar imaging (Supplementary Fig. 3).

3 wild-type mice were used:
1 female, 6 weeks old, was used for in vitro imaging (Fig. 2d).
2 males, 9 weeks old, were used for in vivo imaging (Supplementary Fig. 4 and Supplementary Movie 1).

Wild animals	The study did not involve wild animals.
Field-collected samples	The study did not involve samples collected from the field.
Ethics oversight	All live animal procedures were performed following the National Institutes of Health (NIH) guidelines and were approved by the Institutional Animal Care and Use Committee (IACUC) at the Salk Institute under protocol number 13-00022.

Note that full information on the approval of the study protocol must also be provided in the manuscript.
